# Sensitive electrochemiluminescence (ECL) immunoassays for detecting lipoarabinomannan (LAM) and ESAT-6 in urine and serum from tuberculosis patients

**DOI:** 10.1371/journal.pone.0215443

**Published:** 2019-04-18

**Authors:** Tobias Broger, Michael Tsionksy, Anu Mathew, Todd L. Lowary, Abraham Pinter, Tatiana Plisova, Daniel Bartlett, Simone Barbero, Claudia M. Denkinger, Emmanuel Moreau, Kiyonori Katsuragi, Masanori Kawasaki, Payam Nahid, George B. Sigal

**Affiliations:** 1 FIND, Geneva, Switzerland; 2 Meso Scale Diagnostics, LLC., Rockville, Maryland, United States of America; 3 Department of Chemistry and Alberta Glycomics Centre, University of Alberta, Edmonton, Alberta, Canada; 4 Public Health Research Institute Center, New Jersey Medical School, Rutgers University, Newark, New Jersey, United States of America; 5 Otsuka Pharmaceutical Co., Ltd., Tokyo, Japan; 6 University of California, San Francisco, California, United States of America; University of Cape Town, SOUTH AFRICA

## Abstract

**Background:**

Tuberculosis (TB) infection was responsible for an estimated 1.3 million deaths in 2017. Better diagnostic tools are urgently needed. We sought to determine whether accurate TB antigen detection in blood or urine has the potential to meet the WHO target product profiles for detection of active TB.

**Materials and methods:**

We developed Electrochemiluminescence (ECL) immunoassays for Lipoarabinomannan (LAM) and ESAT-6 detection with detection limits in the pg/ml range and used them to compare the concentrations of the two antigens in the urine and serum of 81 HIV-negative and -positive individuals with presumptive TB enrolled across diverse geographic sites.

**Results:**

LAM and ESAT-6 overall sensitivities in urine were 93% and 65% respectively. LAM and ESAT-6 overall sensitivities in serum were 55% and 46% respectively. Overall specificity was ≥97% in all assays. Sensitivities were higher in HIV-positive compared to HIV-negative patients for both antigens and both sample types, with signals roughly 10-fold higher on average in urine than in serum. The two antigens showed similar concentration ranges within the same sample type and correlated.

**Conclusions:**

LAM and ESAT-6 can be detected in the urine and serum of TB patients, regardless of the HIV status and further gains in clinical sensitivity may be achievable through assay and reagent optimization. Accuracy in urine was higher with current methods and has the potential to meet the WHO accuracy target if the findings can be transferred to a point-of-care TB test.

## Introduction

Tuberculosis (TB), an infection caused by *Mycobacterium tuberculosis* (*Mtb*), was responsible for an estimated 1.3 million deaths in 2017 [1]. As most cases of TB can be safely and effectively treated after early diagnosis, the high observed mortality rate is associated with poor efficiency in identifying affected individuals and delays in treatment initiation [2]. Traditional methods like smear microscopy and nucleic acid tests like Xpert MTB/RIF (Xpert) rely on sputum as the sample matrix, which leads to poorer test sensitivity in certain populations, like people living with HIV and children, who are more likely to have disease that is extra-pulmonary or paucibacillary in nature and less likely to be able to produce sputum [3,4]. Data show that 20–60% of HIV-positive patients presenting for TB diagnosis are unable to produce a sputum sample [5,6].

Diagnosing TB by detection of *Mtb* antigens in blood or urine provides an attractive alternative approach. Antigen detection is also compatible with the use of simple and low-cost immunoassay techniques, like rapid strip tests, that are better suited to the primary care setting in low and middle income countries. Most work to date on antigen-based assays for TB has focused on detection of lipoarabinomannan (LAM), a *Mtb* cell wall lipopolysaccharide. It is well established that LAM can be found in the sputum [7–9] and urine [10–13] of some TB patients. There are also early-stage studies indicating that LAM can also be found in serum [14–19]. Serological approaches based on the detection of serum antibodies to LAM have also been investigated and have found anti-LAM antibodies in many patients, although the serological assays have not provided sufficient accuracy for clinical utility [20].

Commercial implementations of LAM tests have so far been limited to measuring LAM in urine. A commercial lateral flow LAM (LF-LAM) test measuring urinary LAM to diagnose TB is available–the Alere Determine TB LAM Ag test from Abbott Diagnostics–but its adoption has been limited by its poor clinical sensitivity, which a recent meta-analysis estimated at 45% with a 95% confidence range of 29% to 63% [21]. Clinical sensitivity is higher for HIV-positive (HIV+) TB patients with low CD4 counts, so the Alere LF-LAM has been proposed as a complement to the Xpert for immunocompromised HIV+ individuals, potentially compensating for the poorer performance of Xpert in this population [22]. Recently, there has been encouraging evidence that the clinical sensitivity of urinary LAM for diagnosing TB in both HIV- and HIV+ patients can be improved by using advanced assay methods with lower detection limits [23–25] including a high sensitivity test suitable for use at the point-of-care—the SILVAMP TB LAM Ag test—developed by Fujifilm [26].

Relative to LAM, considerably less attention has been given to assays for protein antigens of *Mtb*. A 2011 review of antigen detection assays for TB lists a number of published reports dating back to the 1990s where research assays for undefined TB proteins were used to test a variety of clinical samples [27], but the lack of follow-up work makes it hard to judge the clinical value of these assays. More recently a number of papers have provided evidence that proteins secreted by *Mtb*, or peptides derived from these proteins, may be found in the serum of TB patients when using new sensitive assay techniques. Specific *Mtb* proteins that have been measured in serum or plasma include ESAT-6 [28–33], CFP-10 [28–30,33], Ag85 complex [34–36], Mpt32 and Mpt64 [36] and mshD [37]. Surprisingly, while the preponderance of LAM assays studies use urine as the sample matrix, most studies of *Mtb* protein have focused on blood-derived matrices. One study provided evidence that ESAT-6 can be found in the urine of TB patients, but this study was limited to urine from four subjects [24]. Another study used slow off-rate modified aptamers to assess the diagnostic potential of 11 antigens in serum and urine but found limited diagnostic utility [38]. Finally, an ELISA developed for MoeX detection in urine detected the protein in 44% (11/25) of TB patients [39].

In an earlier study [25], we demonstrated that the application of sensitive electrochemiluminescence (ECL)-based assays and novel antibodies [8,40] to the measurement of LAM could significantly improve the clinical performance of urinary LAM as a diagnostic biomarker for TB relative to the Alere LF-LAM. In a small case-control study including HIV-positive and HIV- negative individuals presenting at care centers with symptoms of TB, the sensitivity for TB detection was 93% over all subjects and 80% when limited to HIV- subjects (in contrast the analogous values for the Alere LF-LAM were 33% and 13%, respectively). In the work reported here, we extend our earlier studies by applying the same approach and clinical cohort to (i) the measurement of LAM in serum and (ii) the measurement of ESAT-6 in both urine and serum. The work is designed to provide a preliminary assessment of the relative clinical utilities of these biomarkers and sample matrices, as well as to provide data that can be used to identify the analytical performance characteristics that will be required for the development of new point-of-care diagnostics targeting these biomarkers.

## Materials and methods

### Antibodies and control materials

Purified LAM from *Mtb* strain Aoyama-B was obtained from Nacalai Tesque Inc. (Japan, Product Number 02449–61). The Aoyama B strain has been commonly used in *Mtb* research and has also been used in Japan for tuberculin production [41]. Recombinant ESAT-6 was obtained from Alpha Diagnostics (San Antonio, TX, USA). *Mtb* culture filtrates were provided by the Biodefense and Emerging Infections Research Resources Repository (BEI). The monoclonal antibodies used in the LAM assays have been previously described [8,25,40,42]. The ESAT-6 assay used monoclonal antibodies from commercial sources (Bioporto, Hellerup, Denmark and MSD, Rockville, MD, USA).

### Immunoassays

Immunoassays for LAM employing a multiplexed sandwich immunoassay format and ECL detection were carried out on commercial instrumentation and multi-well plate consumables from Meso Scale Diagnostics, LLC. (MSD) [43]. The assays were run in MSD’s multiplexed U-PLEX format. The U-PEX format employs multi-well plate consumables, in which each well comprises a screen-printed carbon ink electrode supporting a generic 10-plex array of binding reagents selected for their specificity to a set of 10 U-PLEX “linkers”. Capture antibodies are coupled to these linkers (using reagents available from MSD) to generate reagents that are targeted to specific elements of the generic arrays, enabling the solution phase self-assembly of capture antibody arrays. Running assays in the U-PLEX format involves the steps of (i) incubating a mixture of up to 10 capture antibody-linker conjugates in the U-PLEX wells to self-assemble a capture antibody array and washing to remove excess unbound capture antibody; (ii) adding the sample and a sample diluent comprising blocking components (including blockers of human anti-mouse antibodies), incubating to bind analyte, and then washing to remove unbound sample; (iii) adding labeled detection antibodies, incubating to bind captured analyte and form sandwich complexes, and then washing to remove unbound detection antibody; and (iv) adding an ECL read buffer (MSD Read Buffer Gold) and analyzing the plate on a MSD plate reader. The plate reader applies an electrical potential to the electrode in each well and measures the resulting ECL emission from each array element on the electrode. The LAM assay was run in the U-PLEX format using an array of anti-LAM capture antibodies recognizing a range of different LAM epitopes and a single anti-LAM detection antibody (A194-01). The assay format and instrumentation allowed the assay signal from each capture antibody to be measured independently, although only the results from the two most sensitive capture antibodies (FIND 28 and S4-20) will be presented here. The assay procedure and analytical performance are described in detail in a separate paper [25].

The Immunoassay for ESAT-6 was also conducted using MSD instrumentation and consumables but was carried in a singleplex configuration using MSD’s ultra-sensitive S-PLEX ECL format. The S-PLEX procedure was similar to that described for LAM above, except that instead of using the U-PLEX plates, the capture antibody (Bioporto) was directly immobilized on the carbon ink electrode. In addition, after completion of the detection antibody (MSD) binding step, the S-PLEX format [44–49] includes additional signal enhancement steps using proprietary enhancement reagents to increase assay signal and improve sensitivity.

Quantitation of the target analytes was based on 8-point calibration curves using purified LAM (LAM assays) or *Mtb* culture filtrate (ESAT-6 assay) diluted into a serum-free calibration diluent (2% bovine serum albumin in phosphate buffered saline). Each calibrator concentration was run in duplicate in each assay plate. ESAT-6 concentrations in the *Mtb* culture filtrate were assigned based on comparison to a recombinant ESAT-6 reference material prepared in *E*.*coli*. The relationship of ECL signal to analyte concentration for the calibration curves was fit to a 4-parameter logistic (4-PL) function. Analyte concentrations for test samples were calculated by back-fitting ECL signals to the 4-PL fit and were corrected for sample dilution.

### Sample preparation

To inactivate any host antibodies in urine and serum samples that could interfere with the assay measurement, the samples were heat-inactivated prior to analysis with the LAM or ESAT-6 assays. Serum samples were diluted 1:4 in PBS prior to heat-inactivation to avoid the formation of clots; urine was heat-inactivated without dilution. For heat-inactivation, both, urine and serum samples were heated to 85°C for 10 minutes or 95°C for 5 minutes.

### Clinical samples

For this retrospective case-control study, a total of 75 urine samples and 75 serum samples were selected from FIND’s biobank. For all but six of the urine samples, matching serum samples were available that were collected from the same study subjects on the same day. For the remaining six urine samples (all of which were from TB- HIV+ subjects), serum samples were identified from six additional TB- HIV+ subjects and these were not included in the comparisons that required matched samples. The samples were collected in studies from adults presenting at primary care sites in Bangladesh, Peru, South Africa and Vietnam with clinical symptoms of TB, but not receiving TB treatment at the time of sample collection. Approval by local Ethics Committees and written informed patient consent was obtained before enrolling patients and no personally identifiable information was available to FIND or to the researchers. For use in patient classification, sputum samples (typically two in the first 24 h) were collected from all participants, decontaminated, and tested in up to six independent liquid cultures (MGIT; BD, Franklin Lakes, NJ, USA) and solid cultures (Lowenstein-Jensen medium). The presence of the *M*. *tuberculosis* complex in cultures was confirmed by Ziehl-Neelsen staining or auramine O fluorescence microscopy to identify acid-fast bacilli, MPT64 antigen detection using rapid speciation assays (such as the Capilia TB test; TAUNS, Japan), or molecular methods. TB-positive individuals were patients with at least one positive culture. All TB-positive patients had positive microscopy results. Participants who were smear negative and culture negative on cultures from all sputum samples and who exhibited symptom resolution in the absence of tuberculosis treatment and negative sputum culture results at a 2-month follow-up visit were classified as TB negative. The subjects were further classified as HIV positive or HIV negative on the basis of HIV rapid tests. More details on sample collection and classification of subjects can be found in the description of our previous urinary LAM study [25]. LAM assay results for the urine samples (using the ECL assay and Alere LF-LAM) were available from this previous study. New aliquots of the same urine samples were used for urinary ESAT-6 measurements.

### Data analysis and statistical methods

Data analysis was conducted using the R statistical programming language (version 3.5.1). Confidence intervals for proportions were calculated as the Wilson interval using the “binconf” function from the Hmisc package in R. Comparison of groups was carried out by the Mann-Whitney test using the “wilcox.test” function in R. Correlation constants and p values for the null hypothesis of no correlation were calculated using Pearson’s method and the “cor.test” function in R. Correlations of concentration values were determined using log10 transformed concentrations and only included data points where both methods being compared had detectable concentrations. The Cohen’s kappa statistic for categorical agreement was calculated using the “Kappa” function from the vcd package in R. All relevant data are within the manuscript and its Supporting Information files.

## Results

### Analytical assay performance

**Fig 1:**
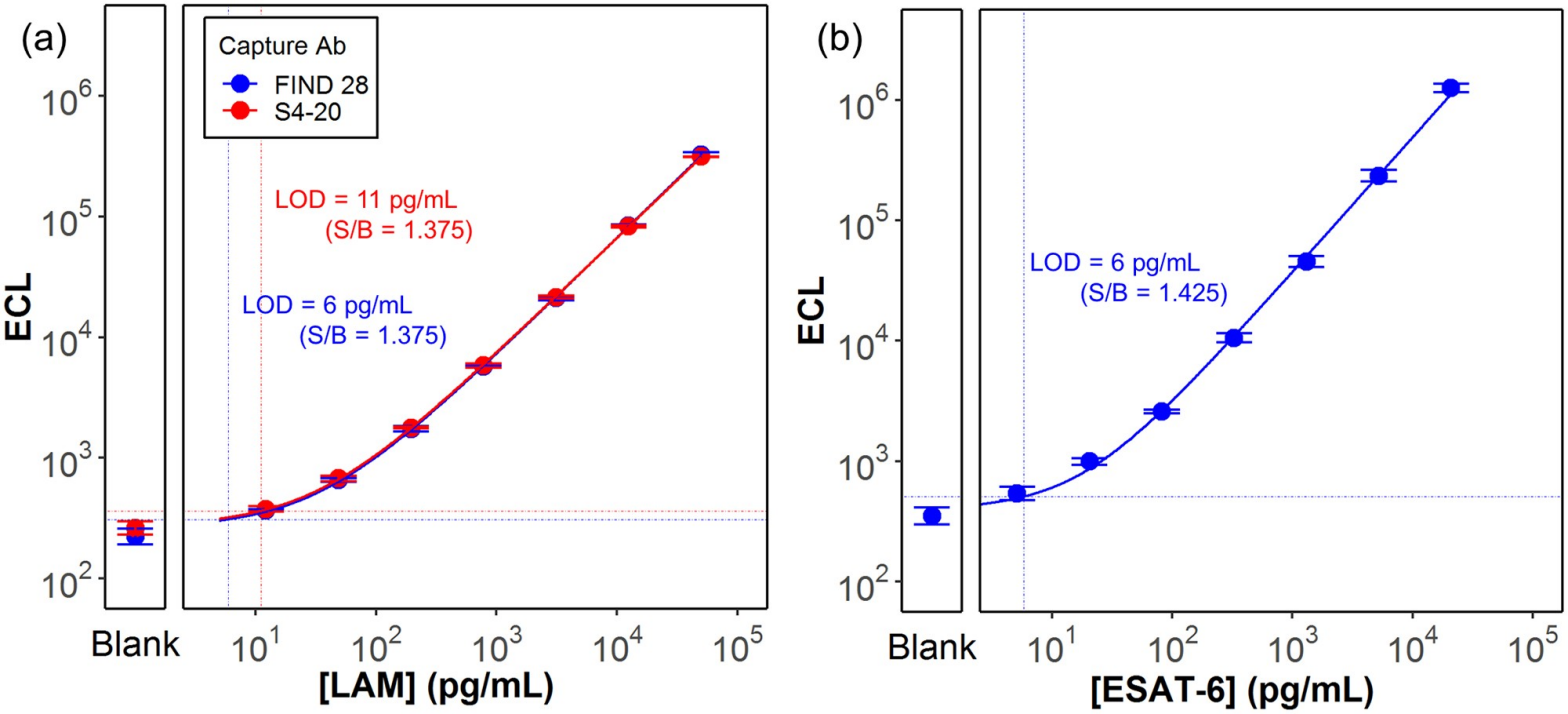
Calibration curves (ECL signal as a function of the concentration) for the (a) LAM and (b) ESAT-6 assays. Each panel also provides the measured signal for a blank sample containing no analyte. Solid lines show the 4PL fit to the data. The horizontal and vertical dashed lines represent, respectively, the lowest detectable signals—based on the threshold signal to background (S/B) value as described in the text—and the limit of detections (LOD) calculated from the lowest detectable signal by back-fitting to the 4PL curve.

The analytical sensitivity of the LAM and ESAT-6 ECL immunoassays was established by testing the LAM and ESAT-6 calibration standards diluted in a serum-free calibration buffer. Fig 1 shows 8 point calibration curves plotting the measured LAM and ESAT-6 assay signals as a function of the concentration of the calibration standards for each assay. For the LAM assay, signals are shown for both the FIND 28 and S4-20 capture antibodies. Based on the Coefficients of variation (CVs) of the blanks and targeting detection thresholds at least 2.5 standard deviations above the blank, we selected a threshold signal-to-blank (S/B) ratio of 1.375 for the LAM assays and 1.425 for the ESAT-6 assay. Average CVs for calibrators above the selected threshold ranged between 3 to 5% for the LAM assays and 9% for the ESAT-6 assay. The calculated analyte concentrations at the selected detection thresholds resulted in limits of detection (LODs) of 6 pg/mL for the ESAT-6 assay, 6 pg/mL for the LAM assay using the FIND 28 capture antibody and 11 pg/mL for the LAM assay using the S4-20 capture antibody (S1 Table).

#### Optimization of sample preparation for urine and serum samples

We examined whether optimal assay performance required sample preparation steps to inactivate proteins in urine or serum that could bind the antigen targets and interfere with antibody recognition. In our previous study of LAM in urine, we found no evidence of inhibitory proteins in urine, but we also found no negative impact on measured LAM concentrations if we heated urine samples under conditions that inactivate host antibodies (85°C for 10 min.). In the current study, we investigated the effect of heat inactivation on detection of LAM in serum and ESAT-6 in serum and urine. S2a Table shows the recovery of LAM spiked at ~3 ng/mL into normal urine or serum samples relative to LAM spiked into a simple buffer (the assay calibrator diluent). The table also shows the improvement in recovery (if any) if the sample was heat inactivated prior to conducting the immunoassay. The measured recoveries in urine in the absence of heat treatment were 63–92% and sometimes higher than after heat treatment confirming our prior observation that matrix inhibition in the urine matrix did not appear to be a significant issue. In contrast, the LAM assays were strongly inhibited by the serum matrix. Heat inactivation of the spiked serum samples largely reversed the matrix inhibition for signals measured with the FIND 28 capture and brought the measured recoveries to 69–100%. Heat inactivation also improved recognition by the S4-20 capture, but to a lesser extent. The effect of heat inactivation was also tested on a small set of urine and serum samples from TB+ individuals (S2b Table). Heat inactivation had minimal effects on the assay signals from urine samples but produced large increases for serum samples. The effect was strongest with the FIND 28 capture, with the signal from one serum sample increasing 45-fold with heat treatment.

In contrast to the LAM assay, significant inhibition of the ESAT-6 assay was not observed when *Mtb* cell culture filtrate containing native ESAT-6 was spiked into either urine or serum to provide a final ESAT-6 concentration of 1 ng/mL (S3a Table). Heat treatment of the spiked samples increased the recoveries to concentrations two to five-fold above the expected values. This increase occurred not only in spiked urine and serum, but also for spiked diluent when compared to an unspiked control. This result suggests that the signal increase is not due to inactivation of interfering species, and is more likely an effect of heating on the ESAT-6 structure likely leading to exposure of epitopes by conformation change such as protein unfolding or by breaking up the association of ESAT-6 with another protein. An increase in signal was also observed after heating of unspiked serum and urine samples from TB+ subjects (S3b Table), although the magnitude of the increase was smaller (1.2-fold to 2-fold). Given the observed stability of ESAT-6 to the heat treatment step, and to protect against the possibility that some fraction of serum samples could contain inhibitory host antibodies, the heat pre-treatment step was made part of the assay protocol for testing clinical samples.

### LAM and ESAT-6 concentrations in clinical samples

Table 1 provides the characteristics of the study population. We selected the samples from FIND’s repository of TB clinical samples to include a range of geographical locations (Asia, Africa and South America), and to cover the different combinations of TB and HIV status. CD4 counts were available for most of the TB+/HIV+ subjects and included subjects above (8 subjects) and below (14 subjects) the 100 cells/uL threshold used in the WHO recommendation for the Alere LF-LAM [22]. Matched serum and urine was available for 69 of the 81 subjects in the study; matched samples were not available for 6 TB-/HIV+ subjects and samples from other TB-/HIV+ subjects was tested to bring the number of serum samples up to 15 in this negative group. Alere LF-LAM was run on all the urine samples and the results were reported with our previous study [25]. The sensitivity of the Alere LF-LAM for the panel of urine samples was 44% (11/25) for HIV+ subjects, but only 13% (2/15) for HIV- subjects, suggesting that the patient population that was tested behaved similarly to the populations described in the Cochrane meta-analysis by Shah et al. [21].

**Table 1:** Characteristics of the study population broken down by TB and HIV status. NA indicates that information for the specified characteristic was not available for a study subject. CD4 cell counts were only available for TB/HIV+ subjects. Alere LF-LAM results were only available for subjects with urine samples. Matched urine and serum samples were available for 69 of the 81 subjects. The remaining 12 subjects consisted of 6 TB+/HIV- subjects with only urine samples and 6 TB+/HIV- subjects with only serum samples.

	All Subjects	TB Negative		TB Positive	
		HIV Negative	HIV Positive	HIV Negative	HIV Positive
**Category**	**No. of Subjects (% of Total)**				
**All subjects**	81 (100%)	20 (25%)	21 (26%)	15 (19%)	25 (31%)
**Gender**					
Female	23 (28%)	6 (7%)	5 (6%)	5 (6%)	7 (9%)
Male	53 (65%)	9 (11%)	16 (20%)	10 (12%)	18 (22%)
NA	5 (6%)	5 (6%)	0 (0%)	0 (0%)	0 (0%)
**Age**					
18 to 40	51 (63%)	6 (8%)	14 (17%)	12 (15%)	19 (23%)
41 to 60	25 (31%)	13 (16%)	6 (7%)	2 (2%)	4 (5%)
61+	2 (2%)	1 (1%)	0 (0%)	1 (1%)	0 (0%)
NA	3 (4%)	0 (0%)	1 (1%)	0 (0%)	2 (2%)
**Location**					
Bangladesh	5 (6%)	5 (6%)	0 (0%)	0 (0%)	0 (0%)
Peru	19 (23%)	3 (4%)	14 (17%)	2 (2%)	0 (0%)
South Africa	20 (25%)	2 (3%)	5 (6%)	5 (6%)	8 (10%)
Vietnam	37 (46%)	10 (12%)	2 (2%)	8 (10%)	17 (21%)
**CD4 Count**					
<= 100 cells/μL	14 (17%)	0 (0%)	0 (0%)	0 (0%)	14 (17%)
> 100 cells/μL	8 (10%)	0 (0%)	0 (0%)	0 (0%)	8 (10%)
NA	59 (73%)	20 (25%)	21 (26%)	15 (19%)	3 (4%)
**Alere**					
Negative	62 (77%)	20 (25%)	15 (19%)	13 (16%)	14 (17%)
Positive	13 (16%)	0 (0%)	0 (0%)	2 (2%)	11 (14%)
NA	6 (7%)	0 (0%)	6 (7%)	0 (0%)	0 (0%)
**Serum and Urine**					
Matched	69 (85%)	20 (25%)	9 (11%)	15 (19%)	25 (31%)

**Fig 2:**
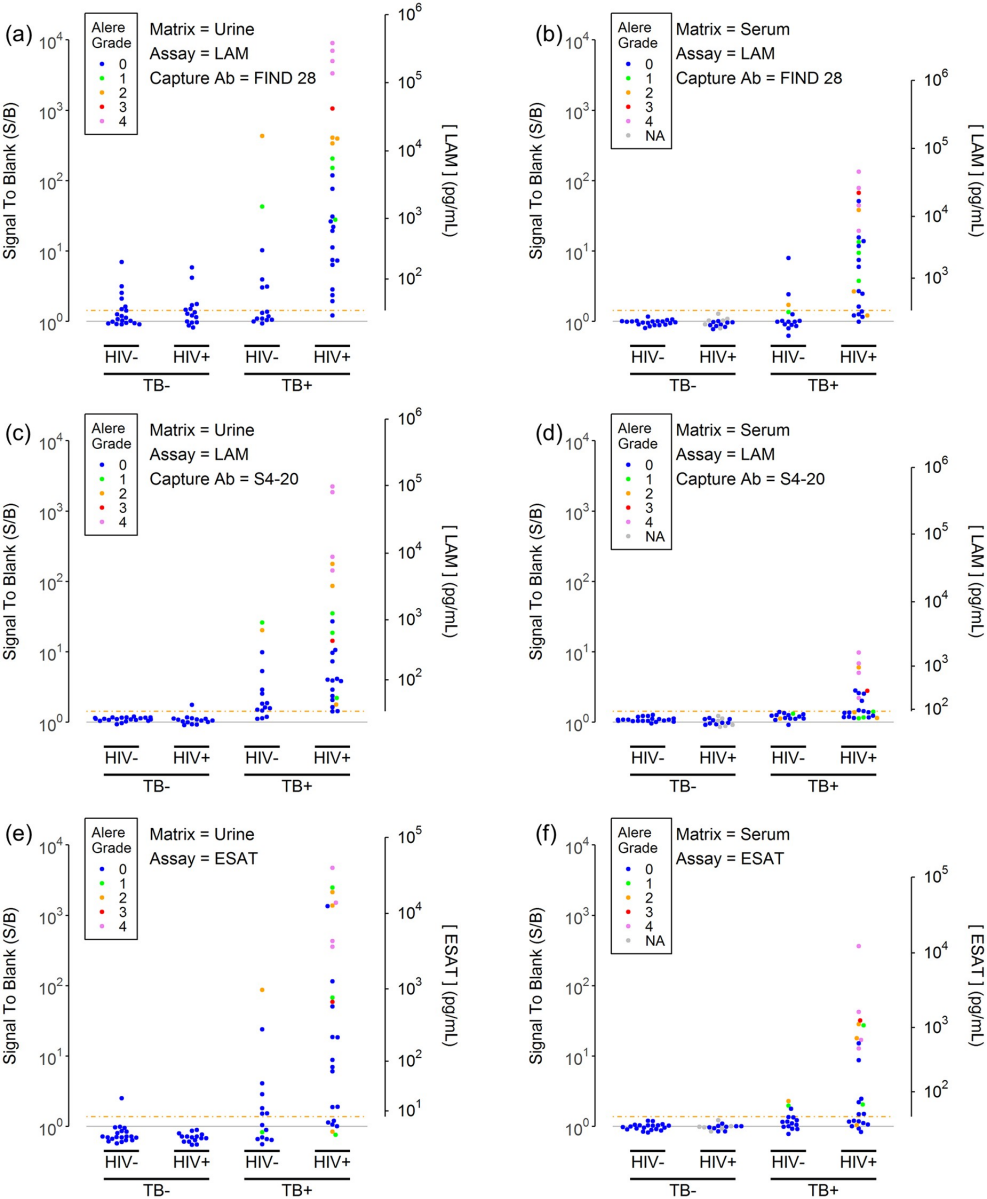
LAM and ESAT-6 concentrations in urine (left column) and serum samples (right column). The plots show LAM (panels a-d) and ESAT-6 (panels e-f) assay signals (normalized to the blank signal) for a set of urine (panels a, c and e) and serum samples (panels b, d and f) as a function of the TB and HIV status of the donor. LAM assay results are provided for the two most sensitive anti-LAM capture antibodies: FIND 28 (panels a-b) and S4-20 (panels c-d). The gray horizontal shows the blank signal (S/B = 1). The dashed orange line shows the assay threshold (S/B = 1.375 for the LAM assays, S/B = 1.425 for the ESAT-6 assay) which is equal to the limit of detection (LOD). The second y axis provides estimated analyte concentrations for concentrations above the LOD. The points are colored by the Alere LF-LAM results for urine from the same subjects (0 = LF-LAM negative, 1 to 4 represents increasing LF-LAM test color intensity as determined by comparison to the Alere LF-LAM reference card).

Fig 2 shows the measured assay signals and measured analyte concentrations for the two LAM assays and the ESAT-6 assay. The results are presented for each of the two sample matrices as a function of TB status and HIV status and are color coded based on urinary Alere LF-LAM test grade. As presented in a previous report [25], the data for LAM concentrations in urine of TB+ subjects ranged over many orders of magnitude, from below the detection limits for the assays (~10 pg/mL) to concentrations greater than 100 ng/mL, with higher concentrations generally being observed for the HIV+ subjects relative to the HIV- subjects. The improved detection limit of the assays led to the detection of LAM in many Alere LF-LAM negative, TB+ urine samples. While the measured signals and LAM concentrations for the assay using FIND 28 as the capture antibody tended to give higher signals than the assay using S4-20 as the capture antibody, the FIND 28 capture also generated a number of false positive results, presumably because of its lower specificity for LAM from *Mtb* [25].

Comparing the previous urinary LAM measurements from our previous work [25] to new results using the two LAM assays to measure LAM in serum, Fig 2 shows that LAM was measurable in serum from at least some TB+ subjects, although the measured concentrations tended to be lower than those in urine. Using the FIND28 capture, the highest concentrations measured in urine and serum were 312 ng/mL and 32 ng/mL, respectively. Similarly to the urine results, concentrations of LAM in serum levels measured with the FIND 28 capture tended to be higher than with the S4-20 capture. The highest concentrations levels measured with FIND 28 and S4-20 were 32 ng/mL and 1.5 ng/mL, respectively. However, in contrast to the urine results, use of the FIND 28 capture did not lead to elevated signals and false positive results for TB- subjects.

Fig 2 also shows that ESAT-6 is directly measurable in urine or serum in several TB+ subjects. Qualitatively, the behavior of ESAT-6 appears similar to LAM, with ESAT-6 concentrations covering roughly the same range as LAM. As observed for LAM, ESAT-6 concentrations tend to be higher in urine vs. serum, and in samples from HIV+ subjects vs. HIV- subjects.

**Fig 3:**
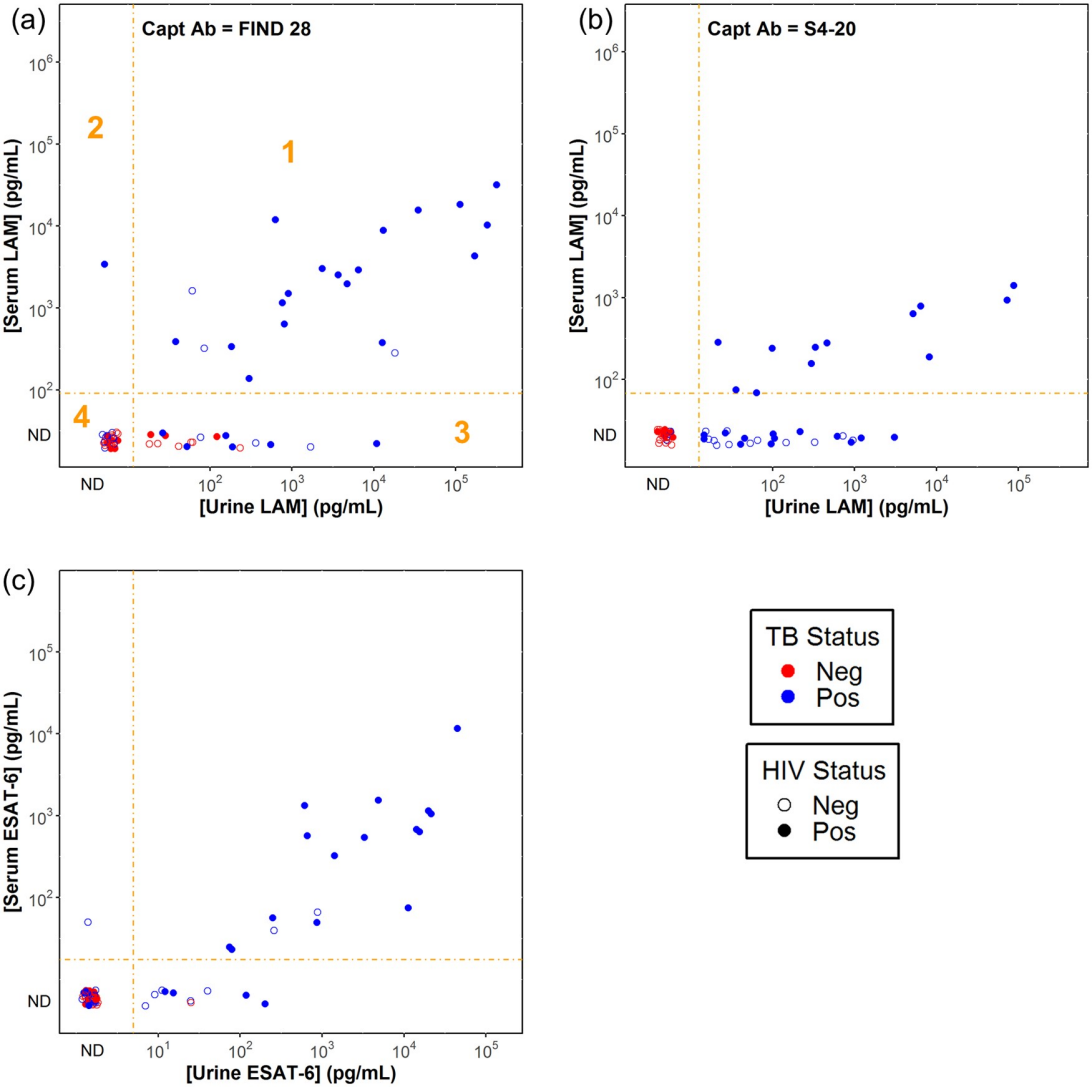
Correlation of LAM and ESAT-6 concentrations in urine and serum. The plots show the results for LAM measurements using the FIND 28 (panel a) and S4-20 (panel b) capture antibodies, and the results for the ESAT-6 assay (panel c). Concentrations in serum are corrected for the additional 4-fold dilution of serum samples relative to urine samples. The dashed orange lines show the dilution-corrected detection limits for the assays. Data points with signals below the detection thresholds (categorized as “ND” or not detectable) are shown for completeness, but do not have assigned concentrations. As indicated with the orange numbering in panel a, the plots can be divided into four regions: (1) concentrations measurable in serum and urine; (2) concentrations only measurable in serum; (3) concentrations only measurable in urine and (4) concentrations not measurable in either matrix. Percentages of categorical agreement are shown in S4 Table. Data points are colored based on TB status and filled based on HIV status as indicated in the key.

Fig 3 shows the correlations between the measured serum and urine concentrations for the subjects with matched serum and urine samples. For the ESAT-6 (Fig 3c) and the FIND 28 LAM assays (Fig 3a), the urine and serum concentrations generally correlated with correlation coefficients of 0.76 (p = 0.0004) and 0.66 (p = 0.001), respectively. Categorical agreement between urine and serum results was 84% and 72% for the ESAT-6 and LAM assays respectively (S4 Table). The concentrations in serum tended to be lower than in urine; the highest ESAT-6 concentrations in urine and serum were 45 ng/mL and 12 ng/ml.

### Clinical assay performance

Table 2 provides the measured clinical sensitivity and specificity across the test sample set for each of the assays, using each of the two sample matrices. The LAM assays using the S4-20 and FIND 28 capture antibodies displayed different relative performance in urine vs. serum. The assay using the S4-20 capture antibody provided better performance for urine samples, primarily due to better sensitivity for the TB+HIV- subjects (80% for S4-20 vs. 40% for FIND 28) and better specificity across all TB- subjects (97% for S4-20 vs. 66% for FIND 28). In contrast, the assay using the FIND 28 capture provided better overall sensitivity for detecting LAM in serum samples from the TB+HIV+ subjects (76% for FIND 28 vs. 48% for S4-20) and TB+HIV- subjects (20% for FIND 28 vs. 0% for S4-20). In addition, the poor specificity of the FIND 28 capture antibody for urine assays was not observed when testing serum samples: both the S4-20 and FIND 28 capture antibodies provided perfect (100%) specificities for testing serum samples regardless of HIV status. As might be expected, given the lowered measured LAM concentrations in serum vs. urine (Figs 2 and 3), even when selecting the optimal capture antibody for the sample matrix, the observed clinical sensitivities for measuring LAM in serum were lower than for LAM in urine.

**Table 2:** Accuracy of LAM and ESAT-6 assays for measurements in urine and serum. LAM performance is provided for the FIND 28 and S4-20 capture antibodies. The table provides the measured sensitivity (correctly classified TB+ samples / total number of TB+ samples) and specificity (correctly classified TB- samples / total number of TB- samples). The values were calculated for the full sample set (All) or for the subsets of samples from HIV- and HIV+ subjects. 95% confidence intervals for the proportion were calculated using Wilson’s method.

Matrix	HIV Status	Assay	Sensitivity		Specificity	
			Correct / Total	% (95% CI)	Correct / Total	% (95% CI)
**Urine**	**All**	**LAM (FIND 28)**	30 / 40	75% (60%-86%)	23 / 35	66% (49%-79%)
	**All**	**LAM (S4-20)**	37 / 40	93% (80%-97%)	34 / 35	97% (85%-100%)
	**All**	**ESAT-6**	26 / 40	65% (50%-78%)	34 / 35	97% (85%-100%)
	**Neg**	**LAM (FIND 28)**	6 / 15	40% (20%-64%)	14 / 20	70% (48%-85%)
	**Neg**	**LAM (S4-20)**	12 / 15	80% (55%-93%)	20 / 20	100% (84%-100%)
	**Neg**	**ESAT-6**	7 / 15	47% (25%-70%)	19 / 20	95% (76%-100%)
	**Pos**	**LAM (FIND 28)**	24 / 25	96% (80%-100%)	9 / 15	60% (36%-80%)
	**Pos**	**LAM (S4-20)**	25 / 25	100% (87%-100%)	14 / 15	93% (70%-100%)
	**Pos**	**ESAT-6**	19 / 25	76% (57%-89%)	15 / 15	100% (80%-100%)
**Serum**	**All**	**LAM (FIND 28)**	22 / 40	55% (40%-69%)	35 / 35	100% (90%-100%)
	**All**	**LAM (S4-20)**	12 / 40	30% (18%-45%)	35 / 35	100% (90%-100%)
	**All**	**ESAT-6**	18 / 39	46% (32%-61%)	35 / 35	100% (90%-100%)
	**Neg**	**LAM (FIND 28)**	3 / 15	20% (7%-45%)	20 / 20	100% (84%-100%)
	**Neg**	**LAM (S4-20)**	0 / 15	0% (0%-20%)	20 / 20	100% (84%-100%)
	**Neg**	**ESAT-6**	3 / 15	20% (7%-45%)	20 / 20	100% (84%-100%)
	**Pos**	**LAM (FIND 28)**	19 / 25	76% (57%-89%)	15 / 15	100% (80%-100%)
	**Pos**	**LAM (S4-20)**	12 / 25	48% (30%-67%)	15 / 15	100% (80%-100%)
	**Pos**	**ESAT-6**	15 / 24	63% (43%-79%)	15 / 15	100% (80%-100%)

The observed clinical sensitivity for the ESAT-6 assay using urine samples (76% for TB+HIV+, 47% for TB+HIV-) was considerably lower than that of the LAM assay using the S4-20 capture antibody (100% for TB+HIV+, 80% for TB+HIV-). The clinical sensitivity for measuring ESAT-6 in serum (63% for TB+HIV+, 20% for TB+HIV-) was lower than that observed using urine samples, but was comparable to the sensitivity of the best serum LAM assay using the FIND 28 capture antibody (76% for TB+HIV+, 20% for TB+HIV-). Good specificity was observed for the ESAT-6 assay in both urine (97%) and serum (100%) when calculated across all subjects regardless of HIV status.

### Comparing and combining LAM and ESAT-6

**Fig 4:**
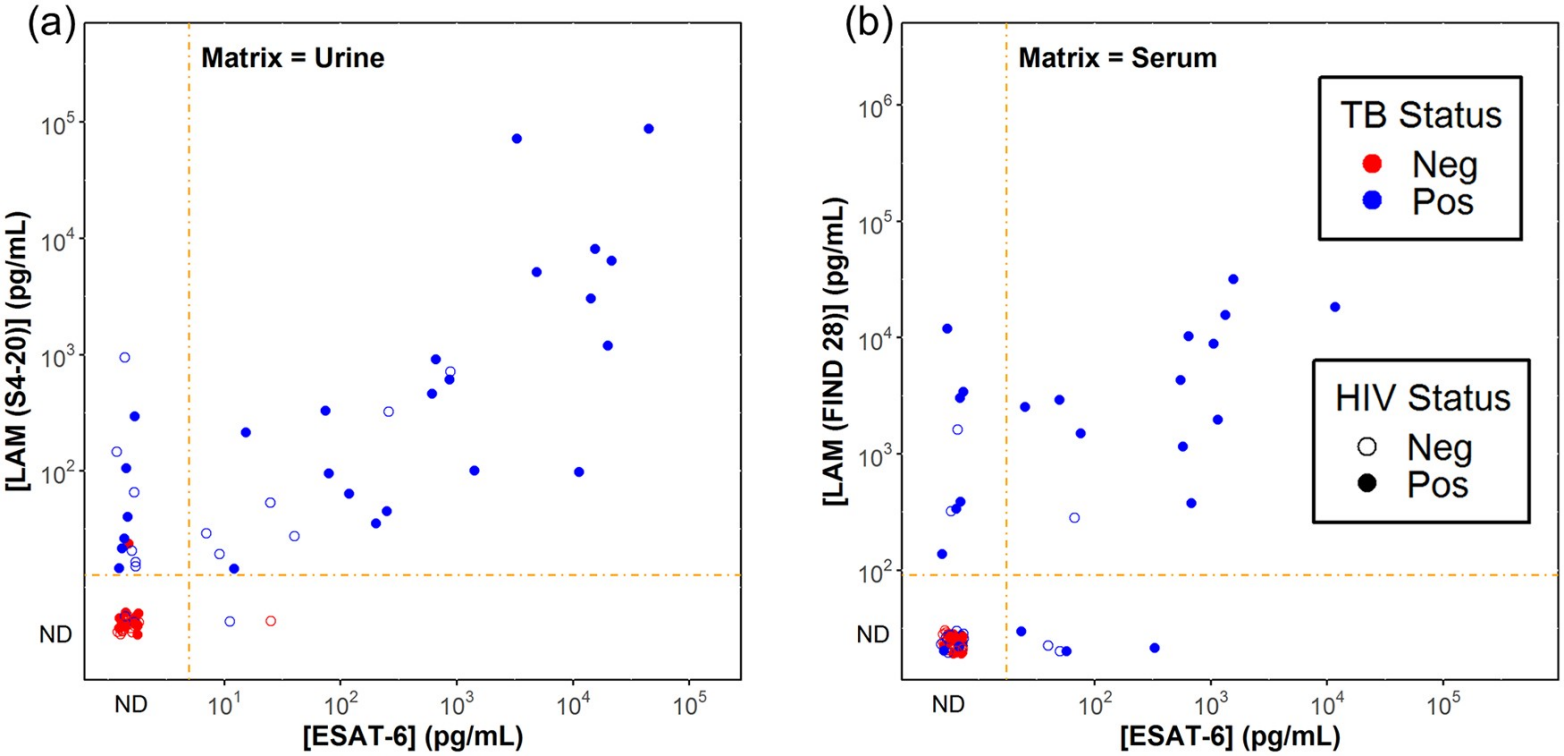
Correlation of LAM and ESAT-6 concentrations. The two plots compare the LAM and ESAT-6 concentrations in urine (panel a) and serum (panel b). The plots display the LAM concentration measured with the most sensitive LAM capture antibody for each matrix: S4-20 for urine and FIND 28 for serum. Concentrations in serum are corrected for the additional 4-fold dilution of serum samples relative to urine samples. The dashed orange lines show the dilution-corrected detection limits for the assays. Data points with signals below the detection thresholds (categorized as “ND” or not detectable) are shown for completeness, but do not have assigned concentrations (see, the discussion in the Fig 3 legend). Percentages of categorical agreement are shown in S5 Table. Data points are colored based on TB status and filled based on HIV status as indicated in the key.

Fig 4 shows the correlation of LAM and ESAT-6 concentrations in urine and serum. The plotted LAM concentrations were determined using the LAM capture antibody with the highest diagnostic accuracy for the matrix: S4-20 for urine and FIND 28 for serum. The correlation coefficients were 0.70 (p < 0.0001) and 0.83 (p < 0.0001) for urine and serum, respectively. The categorical agreement of LAM and ESAT-6 in urine was 80% (S5 Table), the correlation plot for urine samples shows that when ESAT-6 was measurable in urine it tended to track with LAM, and the highest ESAT-6 concentrations were associated with high LAM concentrations. The categorical agreement of LAM and ESAT-6 in serum was 82% (S5 Table). We assessed if sensitivity could be improved by running both assays and using a positive result on either assay to judge a sample as positive (Table 3). Relative to the LAM assay by itself, including the result of the ESAT-6 assay raised the serum sensitivity from 76% to 88% for the TB+HIV+ group and from 20% to 33% for the TB+HIV- group, with no loss in specificity.

**Table 3:** Accuracy of combining ESAT-6 result and LAM result. A sample was characterized as positive if either the LAM or ESAT-6 assays gave signals above their respective thresholds. Sensitivity, specificity and confidence limits were determined as described in the Table 2 legend.

Matrix	HIV Status	Assay	Sensitivity		Specificity	
			Correct / Total	% (95% CI)	Correct / Total	% (95% CI)
**Serum**	**All**	**LAM (FIND 28) + ESAT-6**	26 / 39	67% (51%-79%)	35 / 35	100% (90%-100%)
	**Neg**	**LAM (FIND 28) + ESAT-6**	5 / 15	33% (15%-58%)	20 / 20	100% (84%-100%)
	**Pos**	**LAM (FIND 28) + ESAT-6**	21 / 24	88% (69%-96%)	15 / 15	100% (80%-100%)

#### Effect of immunosuppression on LAM and ESAT-6 concentrations

**Fig 5:**
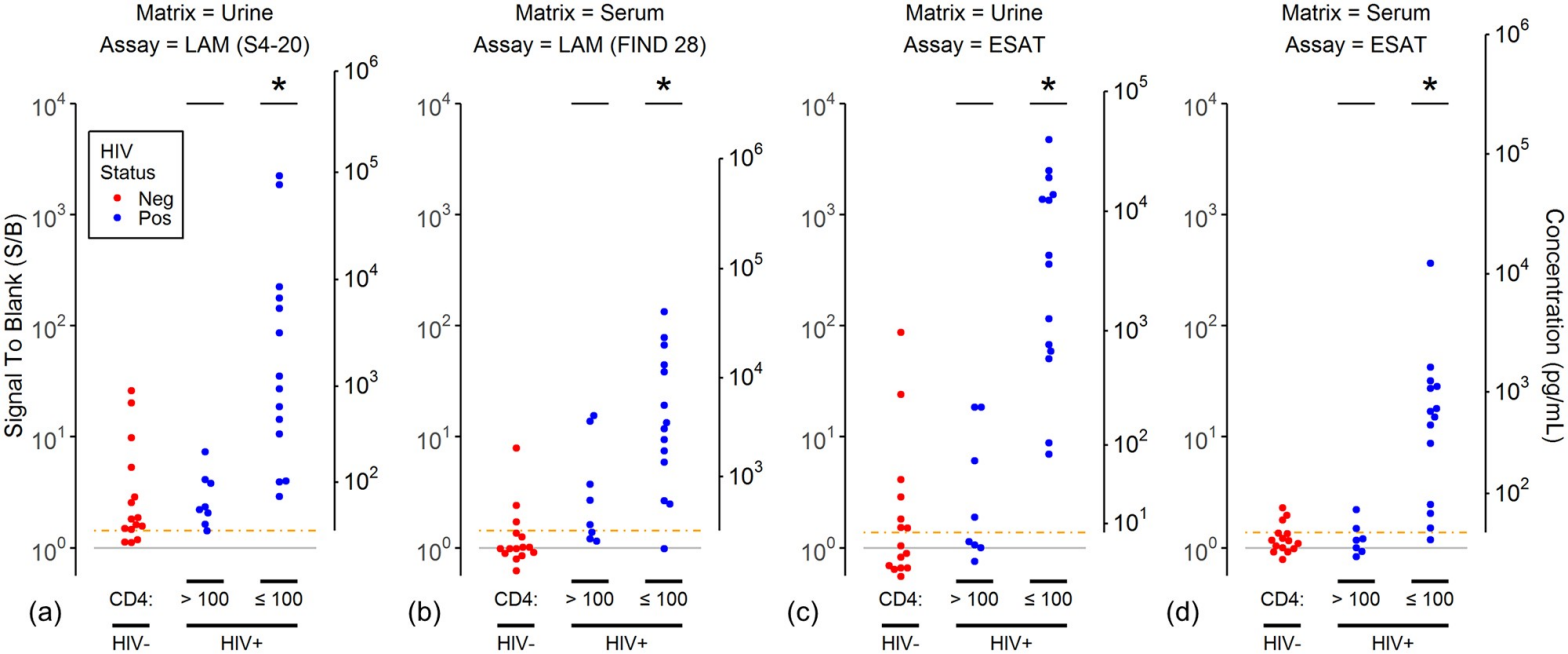
Association of LAM and ESAT-6 concentrations with HIV status and CD4 counts. The plots show the assay signals (left axis) and estimated concentrations (right axis) for (a) LAM measured in urine using the S4-20 capture antibody, (b) LAM measured in serum using the FIND 28 capture antibody, (c-d) ESAT-6 measured in urine and serum. The plots are formatted as described for Fig 2, except only the TB+ subjects are shown, points are colored by HIV status, and the TB+/HIV+ subjects are separated into two groups based on CD4 cell count (> 100 cells/uL and ≤ 100 cells/uL). An “*” above the data points for a TB+HIV+ group indicates the distribution is significantly different than the distribution of points for the TB+HIV- subjects in the same plot (Mann-Whitney test, p < 0.05).

We previously reported that the distribution of LAM concentrations in the urine of immunocompetent TB+/HIV+ subjects was not significantly different from the LAM concentrations of TB+/HIV- subjects but that LAM concentrations were higher in immunosuppressed subjects with CD4 cell counts below 100 cells/uL [25]. We extended this analysis to LAM concentrations in serum and ESAT-6 concentrations in urine and serum (Fig 5). Similar behavior was observed for both bacterial antigens in both matrices. In all cases, the antigen concentrations measured in TB+/HIV- subjects and TB+/HIV+ subjects with CD4 counts greater than 100 cells/uL were not statistically distinguishable, while the distribution of concentrations in TB+/HIV+ subjects with low CD4 counts was shifted significantly to higher values.

## Discussion

In this study, we have shown that LAM and ESAT-6 can be detected in both urine and serum samples of TB patients, using newly developed sensitive ECL immunoassays. The two antigens assayed showed similar concentration ranges within the same sample type. The concentrations were on average roughly 10-fold higher in urine than in serum, but correlated. The measured concentrations of LAM in urine for HIV- patients (from undetectable to about 1,000 pg/mL using the S4-20 capture antibody) were consistent with values reported for this population using a technique involving pre-concentration of LAM from urine using nanocages followed by quantitation by Western blot [24]. It is further known that the Alere LF-LAM threshold is in the range of 1,000 pg/ml which is also consistent with our results.

The measured concentrations of ESAT-6 in serum (0 to about 100 pg/mL for HIV- patients and 0 to about 10,000 pg/mL for HIV+ patients), however, were considerably lower than the concentrations reported by a group using a technique involving trypsin digest of serum samples, concentration of ESAT-6 associated peptides using nanodisks and quantitation by mass spectrometry [30], who reported average concentrations of about 11,000 pg/mL (1.2 nM) in HIV negative TB patients. The reason for this discrepancy is not clear. Such high antigen concentrations are possible in blood-borne diseases; for example, the median concentration of histidine-rich protein II (HRP2) for patients with 1000 to 10,000 malaria parasites/μl blood is reported to be about about 12,000 pg/mL (0.36 nM) [50]. The high concentrations reported by Liu and colleagues seem unlikely for TB patients with pulmonary disease. On the other hand, it is possible that the mass spectrometry technique is picking up ESAT-6 fragments that are not recognized by antibody targeting native ESAT-6. It is also possible that the differences reflect differences in how the assays were calibrated. Standardized control materials and sample panels like those recommended for other disease antigens by the WHO Expert Committee on Biological Standardization [51] should also be developed for TB and would help to resolve these discrepancies.

The correlation between LAM and ESAT-6 suggest that the two antigens enter urine and blood by similar mechanisms in a large fraction of TB patients. Renal infection has been proposed as a mechanism leading to LAM antigenuria [52]. Renal TB is more frequent in immunosuppressed HIV+ and could explain the higher LAM concentrations and higher performance of the Alere LF-LAM in patients with CD4 counts [52]. The authors of this report also argued that the molecular weight of LAM, i.e. when caught in immune-complexes would be too large to pass an intact glomerular basement membrane. We did observe higher concentrations of both LAM and ESAT-6 in the urine of immunosuppressed (CD4 cell counts ≤ 100 cells/uL) TB+HIV+ subjects, relative to TB+HIV- subjects and TB+HIV+ subjects with higher CD4 counts, and renal involvement could play a role for some of those patients. However, by using an assay with about 100-times improved detection limit relative to the Alere LF-LAM, we were able to detect LAM in 80% and ESAT-6 in 47% of urine samples from TB+HIV- subjects (Table 2). These results suggest that the clearance of LAM (or LAM fragments) and ESAT-6 out of blood by the kidneys might play a more important role than originally proposed. Further support for a mechanisms beyond renal infection alone is the finding that both LAM and ESAT-6 can also be detected in serum, and that a large proportion of patients (54% (21/39) for LAM and 63% (17/27) for ESAT-6, S4 Table) with detectable urinary antigen concentrations also have detectable blood antigen concentrations. A limitation of our study was the lack of mycobacterial blood culture and urinary Xpert results, which could have been used to determine if the presence of antigen in blood or urine was associated with blood infections or renal TB.

Binding of LAM to other antibodies and other proteins in clinical sample matrices can potentially interfere with its detection [17]. We observed only minimal urine matrix interference of our LAM assays when measuring LAM spiked into urine and pre-treatment to disrupt potential sequestration of LAM in the urine had little effect on assay signals. In marked contrast, the measurement of LAM spike into serum was almost completely inhibited as has been observed in other LAM studies [15]. We found, however, that this inhibition was almost completely reversed by a simple heat inactivation step using conditions similar to those that have been used to inactivate antibodies in other antigen detection tests [53], and that treatment with acids or proteases pretreatment was not required. We noted that a highly *Mtb* specific capture antibody (S4-20) provided the best clinical performance in urine but had relatively poor sensitivity in serum relative to a more generic anti-LAM antibody (FIND 28). The S4-20 antibody targets a unique capping epitope (MTX-Man, which refers to mannose caps further modified with a 5-methylthio-D-xylofuranose residue) that is almost exclusively found in TB-causing mycobacteria, while FIND 28 targets branched arabinose structures that can be found in LAM from a variety of mycobacteria and related species [8,25,40]. This result could indicate that there are structural differences in the LAM epitopes detected in the two matrices. Interestingly, while the FIND 28 provided poor specificity for urine measurements, it did not provide any false positives in serum, possibly because urine is much more likely to be contaminated with non-*Mtb* commensal organisms that can produce LAM and urine collection is more prone to contamination during sampling. We saw no evidence of matrix inhibition of the ESAT-6 assay in either matrix, although the heat inactivation step appeared to improve the signal generated by ESAT-6, even in the absence of urine or serum. While a mechanism for this effect was not determined, potential explanations could be the exposure of epitopes by conformation change (like protein unfolding) on heating or by disruption of protein complexes.

The preliminary clinical performance results from our case-control study indicate that the ECL LAM and ESAT-6 assays for urine samples have the potential to meet the WHO sensitivity (>65%) and specificity (>98%) targets for a POC TB diagnostic test, providing support for further research and development of improved POC tests targeting these antigens. LAM in urine remains the most promising target. Although the observed clinical sensitivity for detection of LAM and ESAT-6 detection in serum were lower, blood-based tests could play an important role for TB diagnosis in young children, where urine and sputum collection are less feasible.

The clinical performance results presented here provide preliminary evidence for the potential clinical validity of the ECL assays for detecting LAM and ESAT-6 but the generality of the results is limited by the relatively small sample size, the use of a case-control design and the limitation of cases to subjects with smear-positive pulmonary TB. Larger follow-up studies are in progress to better characterize clinical performance through prospective recruitment of relevant and unbiased populations of patients seeking care at TB centers and HIV clinics with symptoms of TB and first results of the Fujifilm SILVAMP TB LAM test are available [26]. It is also important to note that the study was limited to adult subjects, and characterization of performance in a pediatric population is also needed.

Overall, our results suggest that LAM and ESAT-6 are present in urine and serum of TB patients regardless of HIV status. It is difficult to rule out the possibility, particularly for LAM in serum, that there may be forms of LAM present in samples that have epitopes that are either not exposed to or not recognized by our LAM antibodies; further gains in clinical sensitivity may be achievable through assay and/or reagent optimization. The accuracy of blood-based TB tests may also be improved by combining the results of LAM and ESAT-6 measurements, as our results identified subpopulations of TB+ subjects that had detectable concentrations of only one of the antigens. Another option might be the simultaneous detection of the antigen(s) in combination with a host blood marker to come up with a triage test. A recent systematic review indicates that biomarker combinations are more likely to reach the required TPP performance compared to single markers [54]. Research and development in the area of TB antigen detection should be accelerated.

## Supporting information

S1 Table Analytical performance of LAM and ESAT-6 assays. The table summarizes the analytical performance of LAM assays using the 6 different anti-LAM capture antibodies, as well as the performance of the ESAT-6 assay. The results were determined from 8 point calibration curves as described in Fig 1. The first two data columns show signal and CV for the blank sample (n = 10). The third column shows the average CV for calibration standards (n = 4 per level) giving signals above the selected detection signal to blank (S/B) threshold value for each assay (column 4). The last column provides the limit of detection (LOD) calculated as the expected analyte concentration at the threshold as calculated from a 4-PL fit to the calibration curve.
(DOCX)

S2 Table Effect of heat inactivation as a sample pre-treatment step for the LAM assay. (a) Effect of heat inactivation on spike recovery. LAM was spiked into three normal urine samples (Neg Urine), three normal serum samples (Neg Serum) or a simple buffer (Diluent). The concentrations of LAM were measured in each of these samples with each of two LAM capture antibodies (FIND 28 or S4-20), with (Heat) or without (No Heat) pre-treatment of the spiked sample by heat inactivation. The table provides the concentrations normalized to the measured level in diluent without pre-treatment (% Recovery). (b) Effect of heat inactivation on assay signals for samples from TB+ individuals. LAM was measured in three urine samples and three serum samples from TB+ individuals (Pos Urine and Pos Serum). The table provides the assay signals with and without pre-treating the samples with heat inactivation, and also provides the fold-increase in signal with pretreatment (Ratio).
(DOCX)

S3 Table Effect of heat inactivation as a sample pre-treatment step for the ESAT-6 assay. (a) Effect of heat inactivation on spike recovery. ESAT-6 (~ 1 pg.mL) was spiked into three normal urine samples (Neg Urine), three normal serum samples (Neg Serum) or a simple buffer (Diluent). The concentrations of ESAT-6 were measured in each of these samples with (Heat) or without (No Heat) pre-treatment of the spiked sample by heat inactivation. The table provides the concentrations normalized to the measured level in diluent without pre-treatment (% Recovery). (b) Effect of heat inactivation on assay signals for samples from TB+ individuals. ESAT-6 was measured in three urine samples and two serum samples from TB+ individuals (Pos Urine and Pos Serum). The table provides the assay signals with and without pre-treating the samples with heat inactivation, and also provides the fold-increase in signal with pretreatment (Ratio). Note that the samples used to generate this data are not the same samples used to generate the data in S2 Table.
(DOCX)

S4 Table Categorical agreement of urine and serum results. Results are shown for (a) LAM measurements using the FIND 28 capture antibody, (b) LAM measurements using the S4-20 capture antibody, and (c) ESAT-6 measurements. Below each table are point estimates and 95% confidence intervals for the categorical agreement and Cohen’s kappa statistic.
(DOCX)

S5 Table Categorical agreement of LAM and ESAT-6 results for (a) urine and (b) serum. Below each table are point estimates and 95% confidence intervals for the categorical agreement and Cohen’s kappa statistic.
(DOCX)
