# Supplementary material for: Sensitive electrochemiluminescence (ECL) immunoassays for detecting lipoarabinomannan (LAM) and ESAT-6 in urine and serum from tuberculosis patients
Source: PLoS One. 2019 Apr 18;14(4):e0215443. doi: 10.1371/journal.pone.0215443 (PMC6472883; doi:10.1371/journal.pone.0215443)
Supplement: S1 Table — The table summarizes the analytical performance of LAM assays using the 6 different anti-LAM capture antibodies, as well as the performance of the ESAT-6 assay. The results were determined from 8 point calibration curves as described in Fig 1. The first two data columns show signal and CV for the blank sample (n = 10). The third column shows the average CV for calibration standards (n = 4 per level) giving signals above the selected detection signal to blank (S/B) threshold value for each assay (column 4). The last column provides the limit of detection (LOD) calculated as the expected analyte concentration at the threshold as calculated from a 4-PL fit to the calibration curve. (DOCX) [file pone.0215443.s001.docx]

| **Assay (Capture Ab)** | **Blank Signal** | | **Cal Signal** | **Threshold** | **[LOD (pg/mL)** |
| --- | --- | --- | --- | --- | --- |
|  | **ECL** | **ECL CV** | **ECL CV** | **(S/B)** |  |
| LAM (CSU CS-35) | 1,533 | 9% | 5% | 1.375 | 180 |
| LAM (FIND 28) | 224 | 15% | 3% | 1.375 | 6 |
| LAM (Imm 13H3) | 1,128 | 7% | 3% | 1.375 | 210 |
| LAM (Imm 27D2) | 6,492 | 6% | 4% | 1.375 | 2,500 |
| LAM (Otsu S4-20) | 264 | 12% | 3% | 1.375 | 11 |
| LAM (Otsu TB) | 2,062 | 6% | 5% | 1.375 | 59 |
| ESAT (11G4) | 356 | 17% | 9% | 1.425 | 6 |

**S1 Table**. Analytical performance of LAM and ESAT-6 assays. The table summarizes the analytical performance of LAM assays using the 6 different anti-LAM capture antibodies, as well as the performance of the ESAT-6 assay. The results were determined from 8 point calibration curves as described in Figure 1. The first two data columns show signal and CV for the blank sample (n = 10). The third column shows the average CV for calibration standards (n = 4 per level) giving signals above the selected detection signal to blank (S/B) threshold value for each assay (column 4). The last column provides the limit of detection (LOD) calculated as the expected analyte concentration at the threshold as calculated from a 4-PL fit to the calibration curve.
