# Supplementary material for: Sensitive electrochemiluminescence (ECL) immunoassays for detecting lipoarabinomannan (LAM) and ESAT-6 in urine and serum from tuberculosis patients
Source: PLoS One. 2019 Apr 18;14(4):e0215443. doi: 10.1371/journal.pone.0215443 (PMC6472883; doi:10.1371/journal.pone.0215443)
Supplement: S2 Table — (a) Effect of heat inactivation on spike recovery. LAM was spiked into three normal urine samples (Neg Urine), three normal serum samples (Neg Serum) or a simple buffer (Diluent). The concentrations of LAM were measured in each of these samples with each of two LAM capture antibodies (FIND 28 or S4-20), with (Heat) or without (No Heat) pre-treatment of the spiked sample by heat inactivation. The table provides the concentrations normalized to the measured level in diluent without pre-treatment (% Recovery). (b) Effect of heat inactivation on assay signals for samples from TB+ individuals. LAM was measured in three urine samples and three serum samples from TB+ individuals (Pos Urine and Pos Serum). The table provides the assay signals with and without pre-treating the samples with heat inactivation, and also provides the fold-increase in signal with pretreatment (Ratio). (DOCX) [file pone.0215443.s002.docx]

| (a) |  | **% Recovery** | | | |
| --- | --- | --- | --- | --- | --- |
|  |  | **LAM (FIND 28)** | | **LAM (S4-20)** | |
|  | **Sample** | **No Heat** | **Heat** | **No Heat** | **Heat** |
|  | Diluent | 100% | NA | 100% | NA |
|  | Neg Urine 1 | 82% | 87% | 87% | 92% |
|  | Neg Urine 2 | 63% | 57% | 68% | 42% |
|  | Neg Urine 3 | 83% | 78% | 92% | 78% |
|  | Neg Serum 1 | 100% | 100% | 1% | 6% |
|  | Neg Serum 2 | 8% | 69% | 2% | 41% |
|  | Neg Serum 3 | 24% | 76% | 4% | 54% |

| (b) |  | **ECL Signal** | | | | | |
| --- | --- | --- | --- | --- | --- | --- | --- |
|  |  | **LAM (FIND 28)** | | | **LAM (S4-20)** | | |
|  | **Sample** | **No Heat** | **Heat** | **Ratio** | **No Heat** | **Heat** | **Ratio** |
|  | Pos Urine 1 | 70,099 | 77,423 | 1.1 | 16,472 | 21,487 | 1.3 |
|  | Pos Urine 2 | 4,507 | 4,222 | 0.9 | 754 | 703 | 0.9 |
|  | Pos Urine 3 | 580 | 480 | 0.8 | 396 | 290 | 0.7 |
|  | Pos Serum 1 | 322 | 14,434 | 44.9 | 477 | 1,625 | 3.4 |
|  | Pos Serum 2 | 168 | 340 | 2.0 | 209 | 208 | 1.0 |
|  | Pos Serum 3 | 131 | 290 | 2.2 | 188 | 223 | 1.2 |

**S2 Table**. Effect of heat inactivation as a sample pre-treatment step for the LAM assay. (a) Effect of heat inactivation on spike recovery. LAM was spiked into three normal urine samples (Neg Urine), three normal serum samples (Neg Serum) or a simple buffer (Diluent). The levels of LAM were measured in each of these samples with each of two LAM capture antibodies (FIND 28 or S4-20), with (Heat) or without (No Heat) pre-treatment of the spiked sample by heat inactivation. The table provides the levels normalized to the measured level in diluent without pre-treatment (% Recovery). (b) Effect of heat inactivation on assay signals for samples from TB+ individuals. LAM was measured in three urine samples and three serum samples from TB+ individuals (Pos Urine and Pos Serum). The table provides the assay signals with and without pre-treating the samples with heat inactivation, and also provides the fold-increase in signal with pretreatment (Ratio).
