# Supplementary material for: Sensitive electrochemiluminescence (ECL) immunoassays for detecting lipoarabinomannan (LAM) and ESAT-6 in urine and serum from tuberculosis patients
Source: PLoS One. 2019 Apr 18;14(4):e0215443. doi: 10.1371/journal.pone.0215443 (PMC6472883; doi:10.1371/journal.pone.0215443)
Supplement: S3 Table — (a) Effect of heat inactivation on spike recovery. ESAT-6 (~ 1 pg.mL) was spiked into three normal urine samples (Neg Urine), three normal serum samples (Neg Serum) or a simple buffer (Diluent). The concentrations of ESAT-6 were measured in each of these samples with (Heat) or without (No Heat) pre-treatment of the spiked sample by heat inactivation. The table provides the concentrations normalized to the measured level in diluent without pre-treatment (% Recovery). (b) Effect of heat inactivation on assay signals for samples from TB+ individuals. ESAT-6 was measured in three urine samples and two serum samples from TB+ individuals (Pos Urine and Pos Serum). The table provides the assay signals with and without pre-treating the samples with heat inactivation, and also provides the fold-increase in signal with pretreatment (Ratio). Note that the samples used to generate this data are not the same samples used to generate the data in S2 Table. (DOCX) [file pone.0215443.s003.docx]

| (a) |  | **% Recovery** | |
| --- | --- | --- | --- |
|  |  | **ESAT-6** | |
|  | **Sample** | **No Heat** | **Heat** |
|  | Diluent | 100% | 380% |
|  | Neg Urine 1 | 116% | 572% |
|  | Neg Urine 2 | 86% | 224% |
|  | Neg Urine 3 | 117% | 192% |
|  | Neg Serum 1 | 121% | 237% |
|  | Neg Serum 2 | 127% | 272% |
|  | Neg Serum 3 | 123% | 224% |

| (b) |  | **ECL Signal** | | |
| --- | --- | --- | --- | --- |
|  |  | **ESAT-6** | | |
|  | **Sample** | **No Heat** | **Heat** | **Ratio** |
|  | Pos Urine 1 | 66,980 | 136,060 | 2.0 |
|  | Pos Urine 2 | 1,067,414 | 1,839,021 | 1.7 |
|  | Pos Urine 3 | 246,963 | 383,944 | 1.6 |
|  | Pos Serum 1 | 9,991 | 12,089 | 1.2 |
|  | Pos Serum 2 | 6,906 | 8,969 | 1.3 |

**S3 Table**. Effect of heat inactivation as a sample pre-treatment step for the ESAT-6 assay. (a) Effect of heat inactivation on spike recovery. ESAT-6 (~ 1 pg.mL) was spiked into three normal urine samples (Neg Urine), three normal serum samples (Neg Serum) or a simple buffer (Diluent). The levels of ESAT-6 were measured in each of these samples with (Heat) or without (No Heat) pre-treatment of the spiked sample by heat inactivation. The table provides the levels normalized to the measured level in diluent without pre-treatment (% Recovery). (b) Effect of heat inactivation on assay signals for samples from TB+ individuals. ESAT-6 was measured in three urine samples and two serum samples from TB+ individuals (Pos Urine and Pos Serum). The table provides the assay signals with and without pre-treating the samples with heat inactivation, and also provides the fold-increase in signal with pretreatment (Ratio). Note that the samples used to generate this data are not the same samples used to generate the data in Table S2.
