# Supplementary material for: Sensitive electrochemiluminescence (ECL) immunoassays for detecting lipoarabinomannan (LAM) and ESAT-6 in urine and serum from tuberculosis patients
Source: PLoS One. 2019 Apr 18;14(4):e0215443. doi: 10.1371/journal.pone.0215443 (PMC6472883; doi:10.1371/journal.pone.0215443)
Supplement: S4 Table — Results are shown for (a) LAM measurements using the FIND 28 capture antibody, (b) LAM measurements using the S4-20 capture antibody, and (c) ESAT-6 measurements. Below each table are point estimates and 95% confidence intervals for the categorical agreement and Cohen’s kappa statistic. (DOCX) [file pone.0215443.s004.docx]

| **(a) LAM (FIND 28)** | | |  | |
| --- | --- | --- | --- | --- |
|  | |  |  | |
| N = 69 | | Urine LAM+ | | Urine LAM- |
| Serum LAM+ | | 21 | | 1 |
| Serum LAM- | | 18 | | 29 |
|  | |  |  | |
| Categorical agreement | | | 72% (60% - 83%) | |
| Cohen’s kappa | | | 47% (30% - 65%) | |
|  |  | |  | |
| **(b) LAM (S4-20)** | | |  | |
|  | |  |  | |
| N = 69 | | Urine LAM+ | | Urine LAM- |
| Serum LAM+ | | 12 | | 0 |
| Serum LAM- | | 25 | | 32 |
|  | |  |  | |
| Categorical agreement | | | 64% (51% - 75%) | |
| Cohen's kappa statistic | | | 31% (15% - 46%) | |

| **(c) ESAT-6** | |  | |
| --- | --- | --- | --- |
|  |  |  | |
| N = 68 | Urine LAM+ | | Urine LAM- |
| Serum LAM+ | 17 | | 1 |
| Serum LAM- | 10 | | 40 |
|  |  |  | |
| Categorical agreement | | 84% (73% - 92%) | |
| Cohen's kappa statistic | | 64% (46% - 83%) | |

**S4 Table**. Categorical agreement of urine and serum results. Results are shown for (a) LAM measurements using the FIND 28 capture antibody, (b) LAM measurements using the S4-20 capture antibody, and (c) ESAT-6 measurements. Below each table are point estimates and 95% confidence intervals for the categorical agreement and Cohen’s kappa statistic.
