# Supplementary material for: Sensitive electrochemiluminescence (ECL) immunoassays for detecting lipoarabinomannan (LAM) and ESAT-6 in urine and serum from tuberculosis patients
Source: PLoS One. 2019 Apr 18;14(4):e0215443. doi: 10.1371/journal.pone.0215443 (PMC6472883; doi:10.1371/journal.pone.0215443)
Supplement: S5 Table — Below each table are point estimates and 95% confidence intervals for the categorical agreement and Cohen’s kappa statistic. (DOCX) [file pone.0215443.s005.docx]

| **(a) Urine** | |  | |
| --- | --- | --- | --- |
|  |  |  | |
| N = 75 | LAM+ (S4-20) | | LAM- (S4-20) |
| ESAT-6+ | 25 | | 2 |
| ESAT-6- | 13 | | 35 |
|  |  |  | |
| Categorical agreement | | 80% (69% - 88%) | |
| Cohen’s kappa | | 60% (43% - 77%) | |

| **(b) Serum** | |  | |
| --- | --- | --- | --- |
|  |  |  | |
| N = 74 | LAM+ (FIND 28) | | LAM- (FIND 28) |
| ESAT-6+ | 13 | | 5 |
| ESAT-6- | 8 | | 48 |
|  |  |  | |
| Categorical agreement | | 82% (72% - 90%) | |
| Cohen’s kappa | | 55% (33% - 76%) | |

**S5 Table** Categorical agreement of LAM and ESAT-6 results for (a) urine and (b) serum. Below each table are point estimates and 95% confidence intervals for the categorical agreement and Cohen’s kappa statistic.
